# Trihydrophobin 1 Phosphorylation by c-Src Regulates MAPK/ERK Signaling and Cell Migration

**DOI:** 10.1371/journal.pone.0029920

**Published:** 2012-01-06

**Authors:** Weibin Wu, Zhichao Sun, Jingwen Wu, Xiaomin Peng, Huacheng Gan, Chunyi Zhang, Lingling Ji, Jianhui Xie, Haiyan Zhu, Shifang Ren, Jianxin Gu, Songwen Zhang

**Affiliations:** 1 Gene Research Center, Shanghai Medical College, Fudan University, Shanghai, China; 2 Institutes of Biomedical Science, Shanghai Medical College, Fudan University, Shanghai, China; 3 Department of Biochemistry and Molecular Biology, Shanghai Medical College, Fudan University, Shanghai, China; Hungarian Academy of Sciences, Hungary

## Abstract

c-Src activates Ras-MAPK/ERK signaling pathway and regulates cell migration, while trihydrophobin 1 (TH1) inhibits MAPK/ERK activation and cell migration through interaction with A-Raf and PAK1 and inhibiting their kinase activities. Here we show that c-Src interacts with TH1 by GST-pull down assay, coimmunoprecipitation and confocal microscopy assay. The interaction leads to phosphorylation of TH1 at Tyr-6 *in vivo* and *in vitro*. Phosphorylation of TH1 decreases its association with A-Raf and PAK1. Further study reveals that Tyr-6 phosphorylation of TH1 reduces its inhibition on MAPK/ERK signaling, enhances c-Src mediated cell migration. Moreover, induced tyrosine phosphorylation of TH1 has been found by EGF and estrogen treatments. Taken together, our findings demonstrate a novel mechanism for the comprehensive regulation of Ras/Raf/MEK/ERK signaling and cell migration involving tyrosine phosphorylation of TH1 by c-Src.

## Introduction

c-Src is a non-receptor tyrosine kinase and prototype of the Src family kinases (SFKs). Three SFKs, Src, Yes and Fyn, are ubiquitously expressed, whereas the others, including Lyn, Lck, Hck and Blk, are mainly expressed in non-adherent cells of the haematopoietic system. SFKs play key roles in cell proliferation, survival, adhesion, morphology and motility [1], [2]. Src knockout mice die within the first week after birth and show defects in osteoclast development resulting in osteopetrosis [3]. Expression of constitutive active mutant of c-Src promotes cell proliferation and migration [4], [5], [6], [7]. Interestingly, *v*-Src, the viral homologue of the cellular proto-oncogene c-Src encoded by the avian Rous Sarcoma Virus (RSV), is constitutively activated and causes tumor in chickens [8]. Elevated c-Src expression and increased kinase activity have been found in several human cancers and a constitutively activated c-Src mutant was recently identified in human colon carcinoma [9].

The Src family proteins are characterized by four highly conserved Src homology (SH) domains termed SH1 to SH4 [1], [10]. The N-terminal SH4 domain contains a myristoyl group involved in membrane localization. The SH3 and SH2 domains are protein–protein interaction domains, binding with proline rich sequences and phosphotyrosine containing motifs, respectively. A proline-rich linker connects SH2 domain with SH1 domain, the protein kinase domain. The short C-terminal regulatory tail contains Tyr-527, allowing intramolecular interaction with SH2 domain upon phosphorylation [1], [10], [11], [12], [13]. This interaction and binding of linker to SH3 domain stabilize an inactive closed conformation of c-Src. Competitors for SH2 or SH3 domain and dephosphorylation of Tyr527 by protein tyrosine phosphatases (PTP) result in opening of c-Src and partial activation of its kinase activity [2]. Moreover, complete activation of c-Src requires additional autophosphorylation of c-Src at Tyr-416, located in the activation loop of the catalytic centre. Interestingly, phosphorylation of Tyr-527 by the C-terminal Src kinase, CSK, together with dephosphorylation of Tyr-416 by PTP, negatively regulates c-Src kinase activity [13], [14].

The human trihydrophobin 1 (TH1) gene is the homolog of Drosophila TH1, which was originally identified during positional cloning of mei-41 [15], [16]. Further characterization by Bonthron's group demonstrated that the TH1 gene was highly conserved from *Drosophila* to human in sequence comparison, but not found in *Caenorhabditis elegans* [17]. An independent study indicated that TH1 was NELF-C/D, an integral subunit of the human negative elongation factor (NELF) complex [18], which is a four-subunit transcription factor, designated NELF-A, -B, -C/D, and -E in human and Drosophila. NELF complex was reported to associate with RNP polymerase II to induce transcriptional pausing at HSP70 promoter and estrogen receptor response element [19], [20].

Recently, we reported that TH1 served as a negative regulator of A-Raf and PAK1, inhibiting MEK/ERK signaling pathway [21], [22], [23]. Interestingly, TH1 interacts with MEK1 and ERK without directly suppressing their kinase activities [21]. Localized in focal adhesions and filopodia at the leading edge of cells, TH1 inhibits MAPK/ERK-driven cell migration through impacting actin and adhesion dynamics [21]. Moreover, the expression of TH1 negatively correlates with the aggressiveness of human breast cancer cells and breast tumor tissues [24].

In this study, we identified TH1 as an interaction partner of c-Src by immunoprecipitation and GST-pull down assay. Further investigation revealed that c-Src directly phosphorylated TH1 at Y6 *in vivo* and *in vitro*, which led to lowered association with A-Raf and PAK. Dephosphorylated form of TH1 (TH1-Y6F) inhibited c-Src mediated MAPK/ERK pathway activation, whereas phosphorylated form of TH1 did not inhibit. Moreover, c-Src driven cell migration was attenuated by TH1-Y6F over-expression. Induced tyrosine phosphorylation of TH1 was found by EGF and estrogen treatments. In conclusion, we proposed a mechanism for comprehensive regulation of Ras/Raf/MEK/ERK signaling and cell migration involving tyrosine phosphorylation of TH1 by c-Src.

## Results

### c-Src Interacts with TH1 *in vivo* and *in vitro*

**Figure 1:**
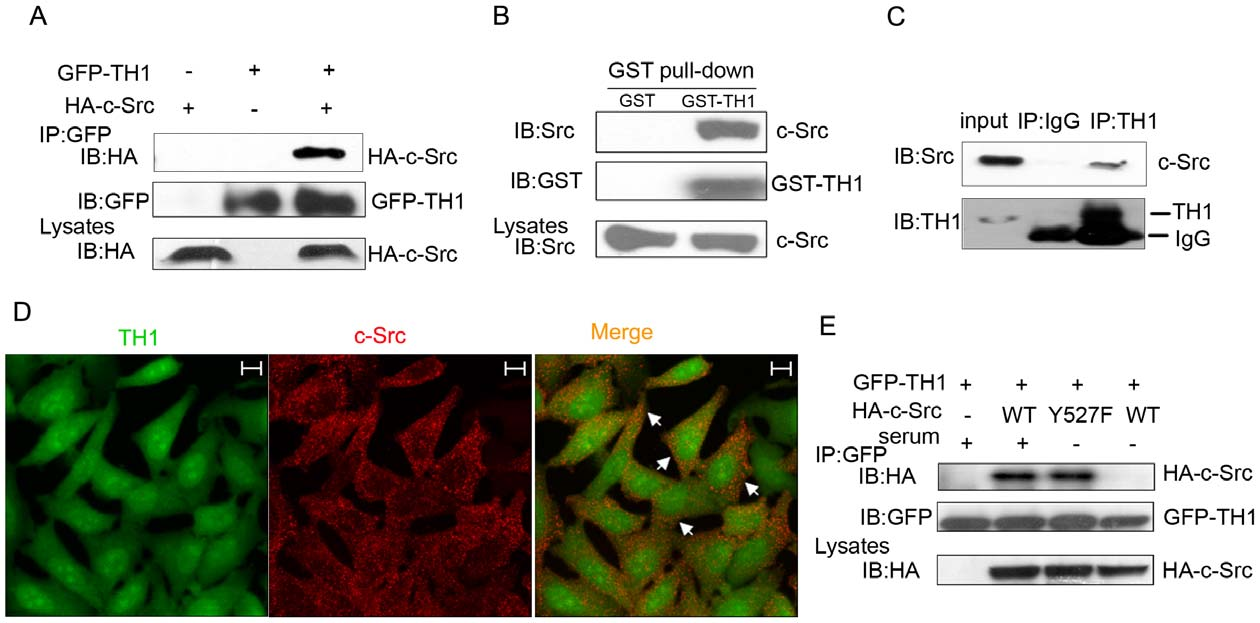
Interaction of c-Src with TH1. (A) c-Src interacts with TH1. HEK293T cells were transiently transfected with GFP-TH1 and/or HA-c-Src as described under “Experimental Procedures”. At 36 h after transfection, the cells were lysed, immunoprecipitated (IP) with 2 µg of anti-GFP antibody, immunoblotted (IB) with an anti-HA antibody. (B) GST-pull down assay detects interaction of c-Src with TH1 *in vitro*. Recombinant GST-TH1 or GST was incubated with HeLa cell lysates, precipitated by glutathione-Sepharose beads. The proteins were subjected to western blot and probed with anti-c-Src and anti-GST antibodies. (C) Interaction of endogenous c-Src with TH1 in HeLa cells. HeLa cells were lysed, immunoprecipitated with rabbit anti-TH1 antibody or control IgG. The proteins were subjected to western blot and probed with anti-c-Src antibody. (D) Colocalization of c-Src with TH1. HeLa cells were stained with anti-TH1 and anti-c-Src antibodies together with FITC-conjugated and Rhodamine-conjugated secondary antibodies, observed under a confocal fluorescence microscope. FITC-TH1 shown in green, Rhodamine-c-Src shown in red, the overlay image shown in yellow, Scale bar: 10 µm. (E) Interaction of TH1 with c-Src mutant under serum deprivation condition. The 293T cells transfected with GFP-TH1 and HA-c-Src mutant were serum starved or stimulated as indicated, immunoprecipitated with anti-GFP antibody, followed by western blot with anti-HA antibody. All the data are representative of at least three independent experiments.

In previous study, we demonstrated that TH1 served as a regulatory protein in MAPK/ERK pathway [21], [22]. To further understand the function of TH1, we performed several co-immunoprecipitation assays, and identified c-Src as an interacting partner of TH1. To confirm the interaction, we cloned c-Src cDNA into HA-tagged expression vector and TH1 cDNA into GFP-tagged expression vector, transfected GFP-tagged TH1 expression vector into HEK293T cells with or without HA-tagged c-Src expression vector. Figure 1A shows that HA-tagged c-Src protein (HA-c-Src) was precipitated by anti-GFP antibodies only when GFP-tagged TH1 was co-expressed. In GST-pull down assay (Figure 1B), GST-TH1 pulled down c-Src from HeLa cell lysates, while GST alone did not, which further confirmed the interaction between c-Src and TH1. As we expected, anti-TH1 antibody precipitated c-Src directly from HeLa cell lysates (Figure 1C), demonstrating endogenous c-Src and TH1 interaction. To analyze the sub-cellular localization of endogenous c-Src and TH1, we immunostained HeLa cells with anti-TH1 and anti-c-Src antibodies and subsequent with FITC-conjugated goat anti-rabbit IgG and Rodamine-conjugated goat anti-mouse IgG secondary antibody. Under confocal microscope, yellow granules were observed in HeLa cells, indicating colocalization of TH1 and c-Src (Figure 1D). Taken together, these data indicate that TH1 interacts with c-Src *in vitro* and *in vivo*.

It has been demonstrated that c-Src stays in a close, inactive state through auto-inhibition without stimuli, switches to an open state upon stimulation, including serum and EGF [1], [10], [11], [12]. Based on the data, we transfected GFP-TH1 into HEK293T cells along with HA-c-Src wild-type(WT) or HA-c-Src active mutant (Y527F) with or without serum for 18 h. Notably, TH1 did not precipitate wild-type c-Src under serum deprivation condition, whereas TH1 precipitated c-Src-Y527F under serum deprivation condition (Figure 1E). These data suggest that the active state is required for c-src interaction with TH1.

### c-Src Phosphorylates TH1 *in vivo* and *in vitro*

**Figure 2:**
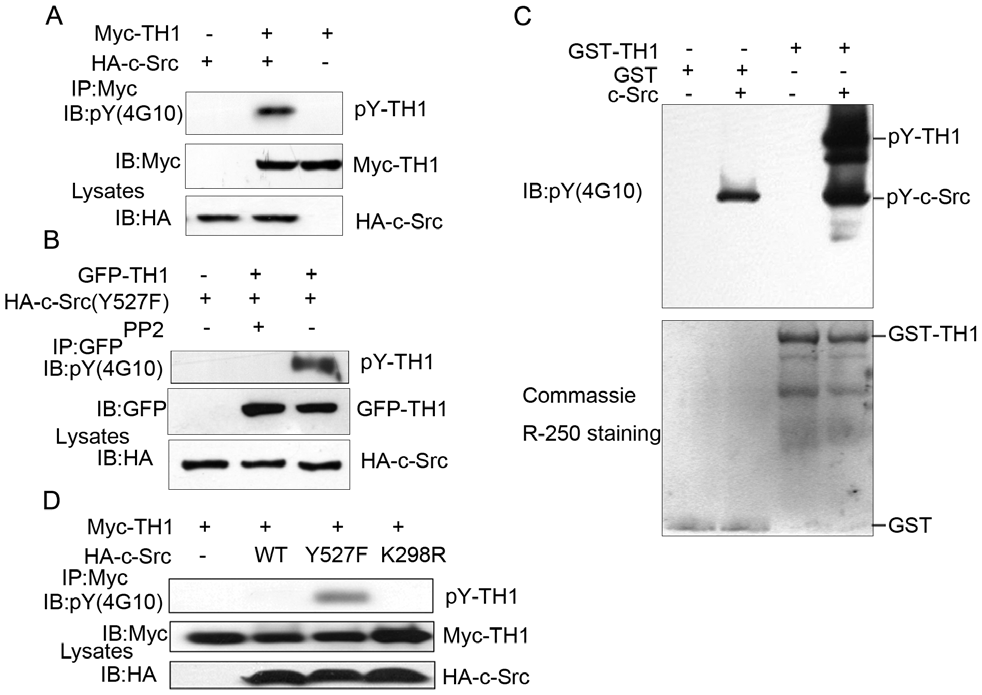
Phosphorylation of TH1 by c-Src. (A) c-Src phosphorylates TH1. COS-7 cells transfected as indicated were lysed, precipitated with anti-Myc antibody bound agarose beads, followed by western blot with anti-phosphotyrosine (4G10) and anti-Myc antibodies. (B) Tyrosine phosphorylation of TH1 by c-Src is abolished by PP2, a specific inhibitor of c-Src. HEK293T cells transfected with GFP-TH1 and HA-c-Src(Y527F) were treated with 10 µM PP2 for 30 min and then immunoprecipitated with anti-GFP antibody, followed by western blot with anti-phosphotyrosine (4G10) and anti-GFP antibodies. (C) c-Src phosphorylates TH1 in vitro. Recombinant GST-TH1 or GST was incubated with recombinant c-Src (5 U) in a kinase buffer as described under “Experimental Procedures”. The reaction mixtures were subjected to western blot and probed with anti-pY (4G10) antibody (upper panel) and then the PVDF membrane was subjected to Coomassie brilliant blue R-250 staining (lower panel). (D) Phosphorylation of TH1 by c-Src mutant under serum deprivation condition. COS-7 cells were transfected with Myc-tagged TH1 and HA-c-Src mutant as indicated, serum-starved for 18 h, lysed and precipitated with anti-Myc antibody bound agarose beads, followed by western blot with anti-phosphotyrosine (4G10) and anti-Myc antibodies. All the data are representative of at least three independent experiments.

To investigate whether TH1 is a *bona fide* substrate of c-Src, we performed in cell phosphorylation assay. COS-7 cells were transfected with Myc-TH1 and HA-c-Src or pcDNA3 empty vector, precipitated by anti-Myc monoclonal antibody, immunoblotted with an anti-phosphotyrosine antibody (4G10). As shown in Figure 2A, tyrosine phosphorylation of TH1 was observed in cells co-expressing HA-c-Src, rather than in other control groups. Furthermore, we utilized PP2, a c-Src-selective tyrosine kinase inhibitor, to determine the specificity of TH1 phosphorylation by c-Src. The results showed that PP2 completely blocked tyrosine phosphorylation of TH1 by c-Src (Y527F) in HEK293T cells (Figure 2B). Next, recombinant TH1 and human c-Src were incubated with ATP for 30 min, which led to significant phosphorylation of GST-TH1 *in vitro* (Figure 2C). Taken together, these data indicate that c-Src phosphorylates TH1 *in vitro* and *in vivo*.

To further investigate whether the active state of c-Src is also required for the phosphorylation of TH1, we transfected various forms of HA-c-Src with Myc-TH1 into COS-7 cells, serum starved for 18 h, followed by detecting tyrosine phosphorylation level of TH1 with the anti-phosphotyrosine antibody. Among three forms, c-Src-Y527F keeps its active state during serum deprivation, while c-Src-WT and c-Src-K298R do not keep their active states during serum deprivation [25]. As shown in Figure 2D, the phosphorylation of TH1 was observed in cells co-expressing c-Src-Y527F, whereas was not observed in cells co-expressing c-Src-WT and c-Src-K298R, suggesting the open, active state of c-Src is also required for the phosphorylation of TH1.

### c-Src Phosphorylates TH1 at Tyr-6

**Figure 3:**
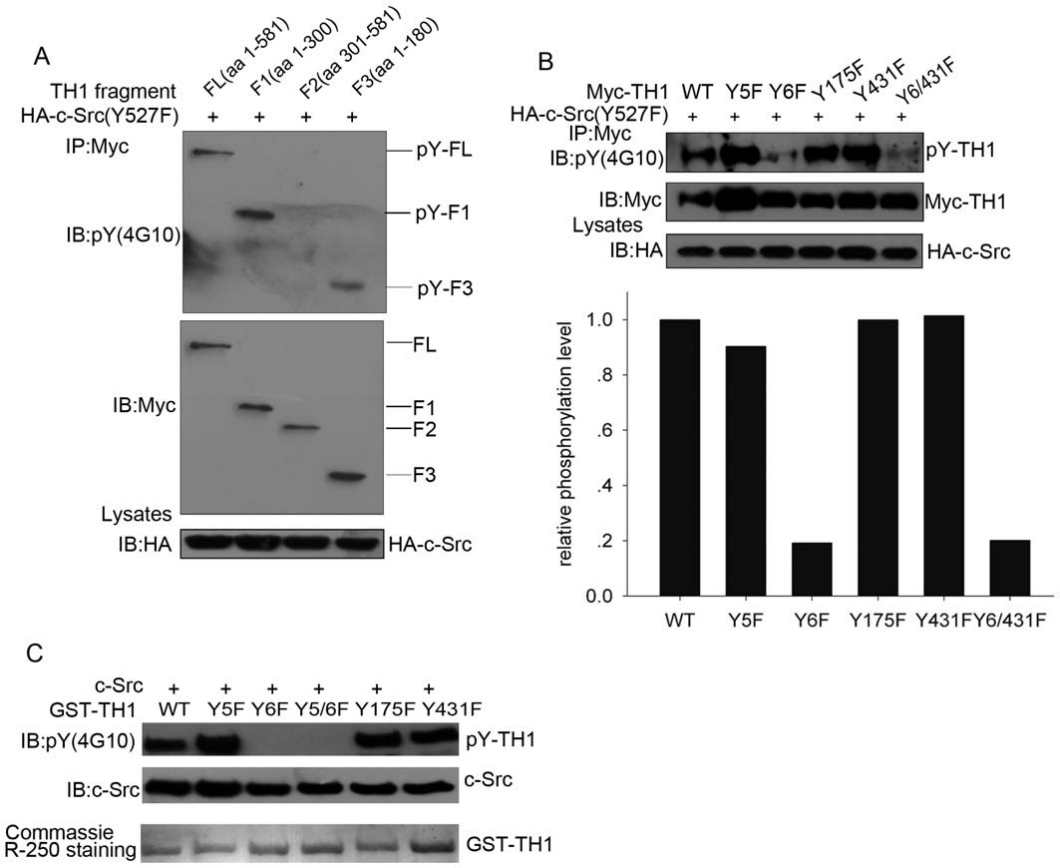
c-Src phosphorylates TH1 at Tyr-6. (A) Phosphorylation of TH1 fragments by c-Src. COS-7 cells were transfected with HA-c-Src (Y527F) and constructs expressing the indicated Myc-tagged TH1 fragments, immunoprecipitated with anti-Myc antibody, followed by western blot with anti-phosphotyrosine and anti-Myc antibodies. (B) Tyr-6 is the major phosphorylation site for TH1. COS-7 cells were transfected with HA-c-Src (Y527F) and constructs expressing the indicated Myc-tagged TH1 mutants, immunoprecipitated with anti-Myc antibody, followed by western blot with anti-phosphotyrosine and anti-Myc antibodies. The quantification of immunoblot intensity is shown at the bottom panel. (C) c-Src phosphorylates TH1 at Tyr-6 in vitro. Recombinant c-Src was incubated with GST-TH1 or GST-TH1 mutant as indicated at 30°C for 30 min and then subjected to western blot and probed with anti-phosphotyrosine and anti-c-Src antibodies. Coomassie brilliant blue staining showed the input of GST-TH1 mutant proteins. All the data are representative of at least three independent experiments.

To determine the phosphorylation site of TH1, we constructed 3 Myc-tagged fragments of TH1: FL, full length (residue 1–581); F1, N-terminal domain (residue 1–300) and F2, C-terminal domain (residue 301–581). Next, we transfected HA-c-Src(Y527F) into COS-7 cells with Myc-tagged TH1 fragments, precipitated with anti-Myc antibody and detected phosphorylation of TH1 with anti-phosphotyrosine antibody. As shown in Figure 3A, c-Src phosphorylated both full length and N-terminal TH1, except C-terminal TH1. To further narrow down the phosphorylation site, we constructed F3, a short N-terminal domain (residue 1–180). The results showed that F3 was sufficiently phosphorylated by c-Src (Figure 3A), indicating c-Src phosphorylation sites were located within N-terminal residue 1–300, possibly within 1–180 of TH1. We then utilized Netphos 2.0 (http://www.cbs.dtu.dk/services/NetPhos/) to predict possible phosphorylation sites within TH1 and got 4 potential phosphorylation sites: Y5, Y6, Y175 and Y431. Based on this, TH1 point mutants including single mutants (Y5F, Y6F, Y175F and Y431F) and a double mutant (Y6/431F), generated by replacing Tyrosine with Phenylalanine, were transfected into COS-7 cells along with c-Src (Y527F). After precipitation, phosphorylation of TH1 mutants was measured by the anti-phosphotyrosine antibody (4G10). Figure 3B showed that the single mutant Y6F and the double mutant Y6/431F were poorly phosphorylated, while the wild type TH1 and the other TH1 mutants were strongly phosphorylated, suggesting Tyr-6 is the major phosphorylation site. To further confirm phosphorylation of TH1 at Tyr-6 in vitro, various TH1 mutants, including Y5F, Y6F, Y5/6F, Y175F and Y431F, were fused to GST, constructed into pGEX-4T-1 vector, expressed in E. coli, purified and incubated with recombinant active c-Src as described in Material and Methods. Figure 3C showed that single mutant Y6F and double mutant Y5/6F were not phosphorylated, while the wild type TH1 and the other TH1 mutants were strongly phosphorylated *in vitro*. Taken together, these data indicate that c-Src phosphorylates TH1 at Tyr-6.

### Tyr-6 Phosphorylation of TH1 Decreases Its Inhibition on MAPK/ERK Signaling

**Figure 4:**
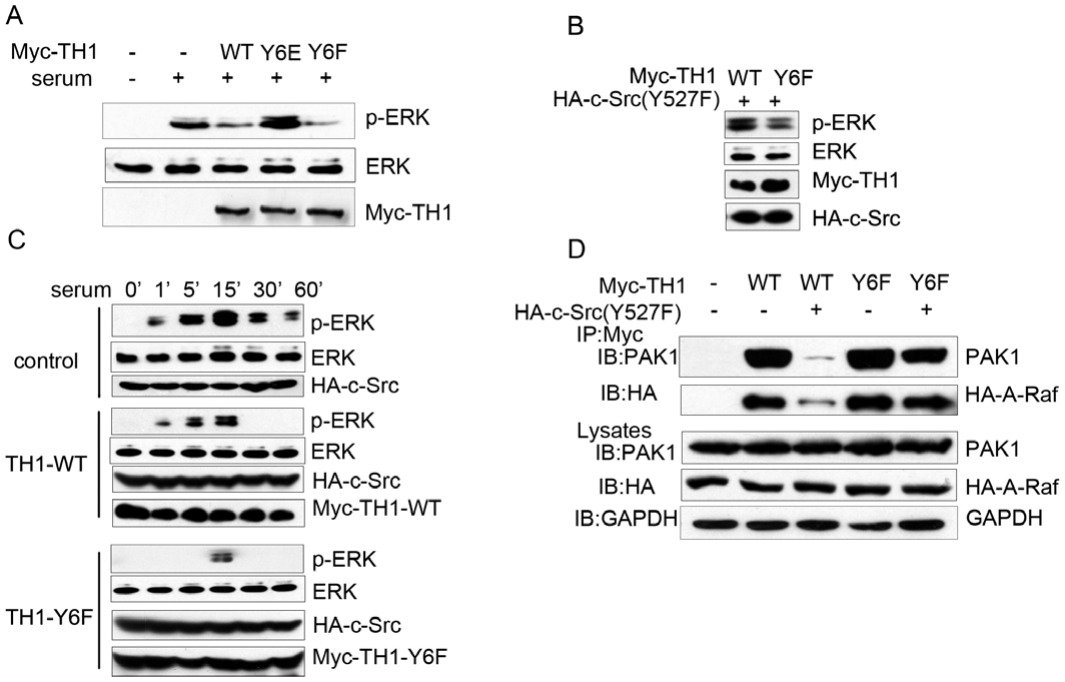
Tyr-6 phosphorylation of TH1 impairs its inhibition on MAPK/ERK signaling. (A) TH1-Y6E mutant lost its inhibition on ERK phosphorylation. HeLa cells were transfected either with Myc-TH1-WT (wild type of TH1), with Myc-TH1-Y6E (the constitutive phosphorylation form of TH1), or with TH1-Y6F (the dephosphorylation form of TH1) and then serum starved for 18 h, stimulated with or without 20% serum for 15 min, lysed, followed by western blot with antibodies against phospho-ERK, total ERK and Myc. (B) TH1-Y6F mutant shows increased inhibition on ERK phosphorylation. HeLa cells transfected with Myc-TH1-WT or Myc-TH1-Y6F were cotransfected with HA-c-Src (Y527F), lysed, followed by western blot with antibodies against phospho-ERK, total ERK, Myc and HA. (C) Time course of ERK phosphorylation. HeLa cells transfected either with Myc-TH1-WT, or with Myc-TH1-Y6F were cotransfected with HA-c-Src, serum starved for 18 h, stimulated with 20% serum for the indicated times. Cell lysates were subjected to western blot with antibodies against phospho-ERK, total ERK, Myc and HA. (D) Phosphorylation of TH1 by c-Src decreases its interaction with PAK1 and A-Raf. HeLa cells transiently transfected with HA-A-Raf and Myc-TH1-WT or Myc-TH1-Y6F were cotransfected with c-Src (Y527F) or control vector. At 36 h after transfection, cell lysates were immunoprecipitated with anti-Myc antibodies. Myc–TH1 associated HA-A-Raf or PAK1 was monitored by anti-HA or anti-PAK1 antibody in the following western blot analyses. Whole cell lysates were also subjected to western blot with anti-HA and anti-PAK1 antibodies. All the data are representative of at least three independent experiments.

To investigate biological impact of TH1 phosphorylation, we constructed two Y6 mutant derivatives, Y6E (replacing Tyrosine with Glutamic acid) to mimic constitutive phosphorylation and Y6F to abolish phosphorylation. Our previous study demonstrated that TH1 played an inhibitory role in MAPK/ERK signaling [21], [24]. Therefore, we examined whether phosphorylation of TH1 played a role in MAPK/ERK signaling. HeLa cells were transfected with Myc-tagged various forms of TH1 (wild type TH1, TH1-Y6E and TH1-Y6F), serum starved for 18 h, stimulated with or without serum for 15 min. Phosphorylation of ERK was measured by an antibody against phospho-ERK. Consistent with previous results, wild type TH1 inhibited ERK phosphorylation (Figure 4A). However, this inhibition of TH1 on ERK phosphorylation was mostly abolished in TH1-Y6E, the constitutive phosphorylation form of TH1 (Figure 4A), suggesting Tyr-6 phosphorylation of TH1 decreases its inhibition on MAPK/ERK signaling.

It has been demonstrated that constitutive active c-Src triggers sustained activation of ERK signaling [26], [27]. To further investigate the impact of TH1 phosphorylation on MAPK/ERK signaling, we transfected HA-c-Src (Y527F) into HeLa cells with TH1 or TH1-Y6F (the dephosphorylated form of TH1), and measured ERK phosphorylation. Compared with TH1-WT, TH1-Y6F further decreased ERK phosphorylation driven by active c-Src (Y527F) (Figure 4B). We next investigated the time course of inhibition of ERK phosphorylation by TH1-WT and TH1-Y6F. HeLa cells over-expressed HA-c-Src were transfected with control vector, TH1-WT and TH1-Y6F respectively, serum starved for 18 h, and then stimulated with serum for 0, 1 min, 5 min, 15 min, 30 min and 60 min. After stimulation, ERK phosphorylation was measured. As expected, TH1-WT inhibited ERK phosphorylation driven by c-Src (Figure 4C). Interestingly, TH1-Y6F further inhibited ERK phosphorylation and delayed ERK phosphorylation until 15 min (Figure 4C).

Our previous study showed that TH1 interacted with A-Raf and PAK1. Through interaction, TH1 inhibited their kinase activities and attenuated MAPK/ERK signaling [21], [22]. To determine whether phosphorylation of TH1 impacts its interaction with A-Raf and PAK1, we transfected HA-A-Raf, Myc-TH1-WT, Myc-TH1-Y6F and c-Src (Y527F) into HeLa cells as indicated in Figure 4D, measured TH1 association with A-Raf and PAK1. Without c-Src over-expression, TH1-WT and TH1-Y6F precipitated similar amount of PAK1 and A-Raf. Interestingly, under c-Src over-expression, TH1-WT precipitated much less amount of PAK1 and A-Raf when compared with TH1-Y6F, the dephosphorylated form of TH1 (Figure 4D). It has been demonstrated that the association between TH1 and A-Raf or PAK1 was important for TH1 to regulate their kinase activities [21], [22]. Thus, Tyr-6 phosphorylation of TH1 by c-Src decreases its interaction with A-Raf and PAK1, releases A-Raf and PAK1 to perform their biological functions, which may explain its decreased inhibition on MAPK/ERK signaling.

### Tyr-6 Phosphorylation of TH1 Impacts Its Stability

**Figure 5:**
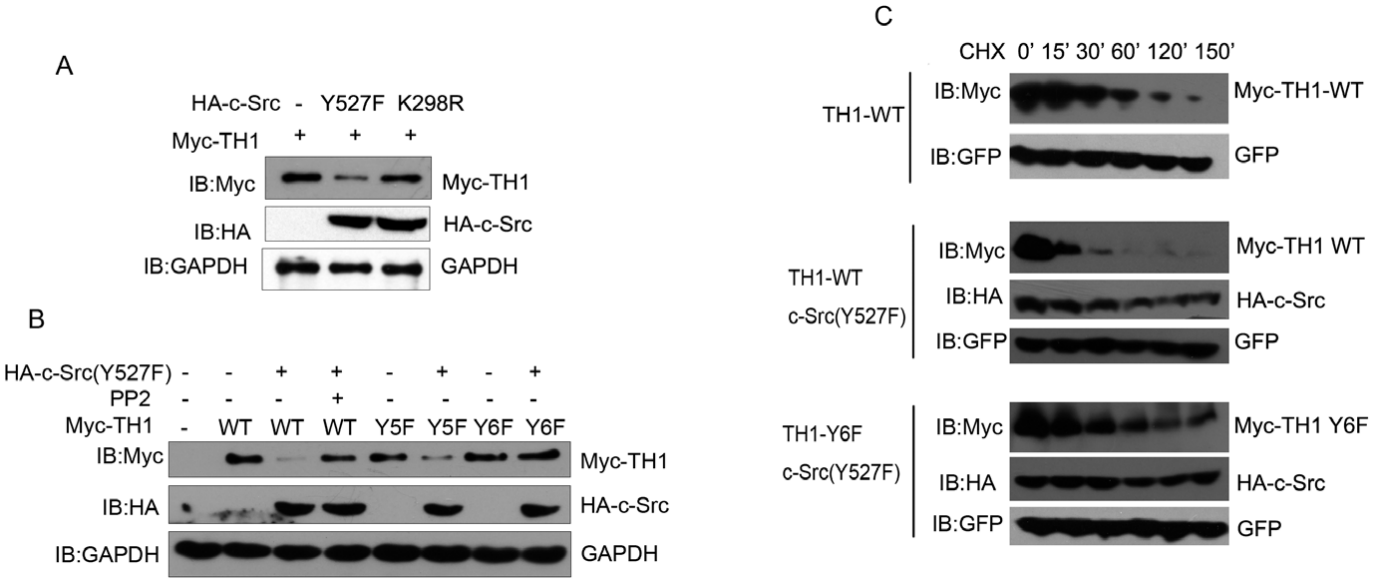
Tyr-6 phosphorylation of TH1 impacts its stability in HeLa cells. (A) c-Src (Y527F) lowers TH1 expression level. HeLa cells transiently transfected either with HA-c-Src (Y527F), with HA-c-Src (K298R), or with control vector were cotransfected with Myc-TH1 for 36 h, pretreated with 100 µM pervanadate, lysed, subject to western blot with antibodies against Myc, HA and GAPDH as internal control. (B) Both PP2 treatment and TH1-Y6F mutant inhibit c-Src mediated TH1 lowering. HeLa cells transfected with Myc-tagged TH1 mutant and HA-c-Src(Y527F) or control vector as indicated for 36 h were treated with 10 µM PP2 or vehicle for 1 h, pretreated with 100 µM pervanadate, lysed, subjected to western blot with antibodies against Myc, HA and GAPDH as internal control. (C) Time course of TH1 degradation. HeLa cells transfected with Myc-TH1-WT or Myc-TH1-Y6F and GFP expression vector (GFP level was used as transfection control) were cotransfected with HA-c-Src (Y527F) or control vector as indicated for 36 h, treated with 50 µg/ml cycloheximide (CHX) to inhibit protein synthesis, and 100 µM pervanadate to inhibit dephosphorylation. Lysates were harvested at the time points as indicated, subjected to western blot with antibodies against Myc and HA.

We next determined whether Tyr-6 phosphorylation of TH1 would impact its stability. As shown in Figure 5A, c-Src (Y527F) over-expression lowered protein expression level of TH1, whereas, c-Src (K298R) (the dominant negative mutant of c-Src [25]) did not decrease. To further confirm the specificity of c-Src in this inhibition, we utilized PP2, a c-Src inhibitor, and found that PP2 treatment blocked c-Src (Y527F) mediated TH1 lowering (Figure 5B). As expected, the dephosphorylation form of TH1, TH1-Y6F, was resistant to c-Src (Y527F) mediated TH1 lowering, whereas, another un-relevant point mutation TH1-Y5F was not resistant (Figure 5B).

To address whether c-Src (Y527F) driven TH1 lowering is mediated through decreased TH1 stability, we treated HeLa cells with cycloheximide (CHX), an inhibitor of protein synthesis, investigated the time course of TH1 degradation. Figure 5C showed that c-Src (Y527F) over-expression promoted degradation of TH1 evidently. Interestingly, the dephosphorylated form of TH (TH1-Y6F) was resistant to c-Src mediated TH1 degradation. Moreover, half life of TH1-Y6F in the presence of c-Src (Y527F) over-expression is longer than that of TH1-WT under the normal condition without c-Src over-expression (Figure 5C lower panel vs. upper panel). Taken together, these data indicate that tyrosine phosphorylation of TH1 at Y6 impacts its stability.

### Enhanced Cell Migration through Tyr-6 Phosphorylation of TH1

**Figure 6:**
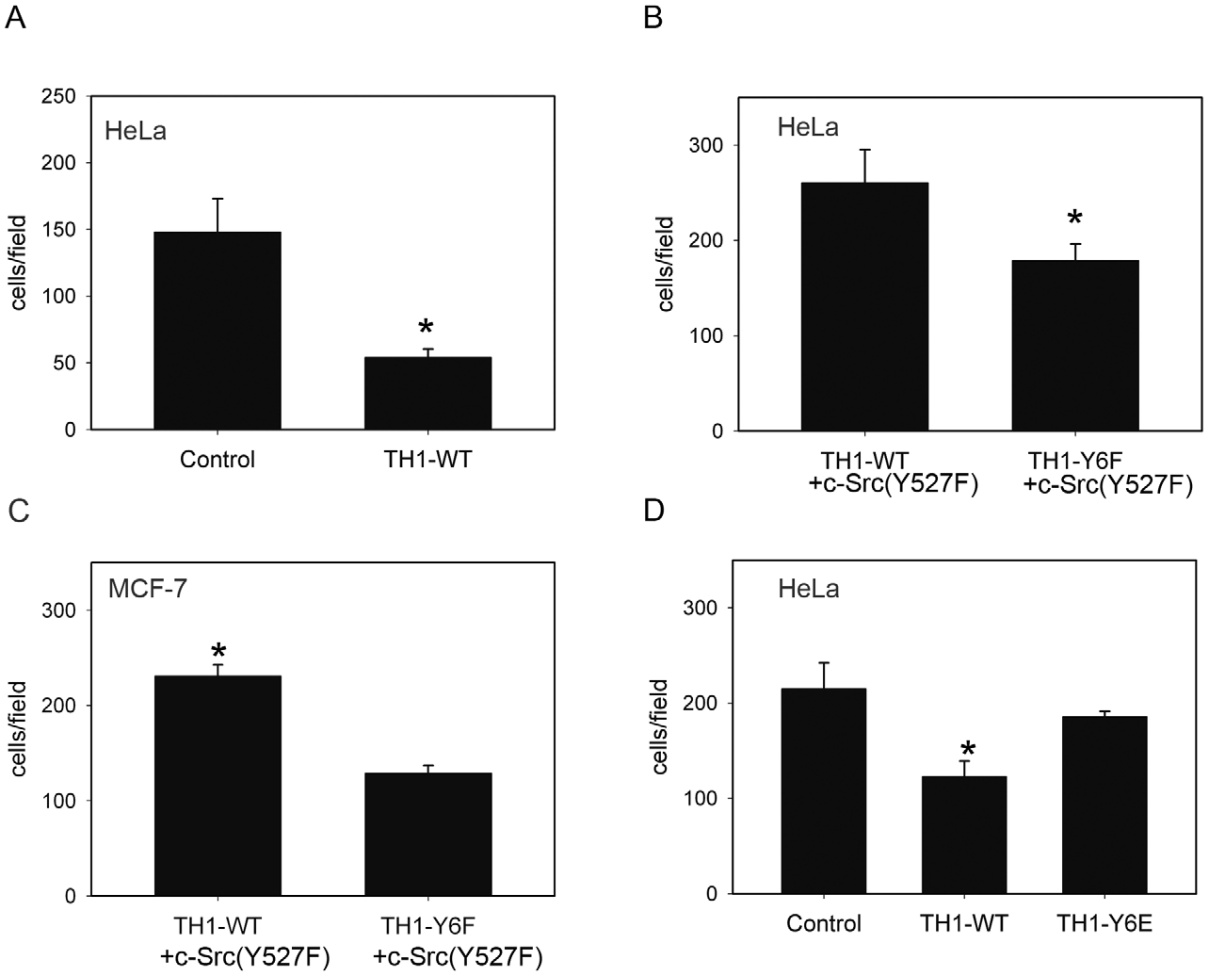
Enhanced cell migration through Tyr-6 phosphorylation of TH1. (A) TH1-WT inhibits HeLa cell migration. HeLa cells were transfected with Myc-TH1-WT or control vector. Transwell cell migration assay was performed as described under “Experimental Procedures” (n = 4, mean ± SD, P<0.01 vs. Control). Statistical significance was determined by the Student's t test. (B–C) TH1-Y6F mutant shows increased inhibition on HeLa and MCF-7 cells migration. HeLa and MCF-7 cells transfected with Myc-TH1-WT or Myc-TH1-Y6F were cotransfected with HA-c-Src (Y527F) and then measured migration by Transwell cell migration assay (n = 3, mean ± SD, P<0.05 TH1-WT vs. TH1-Y6F). (D) Inhibition of cell migration is abolished in TH1-Y6E mutant. HeLa cells were transfected with Myc-TH1-WT, Myc-TH1-Y6E or control vector, and then measured migration by Transwell cell migration assay (n = 4, mean ± SD, P<0.01 TH1-WT vs. Control, P = 0.2 TH1-Y6F vs. Control).

Ectopic expression of TH1 was reported to affect cell adhesion dynamics, which plays a critical role in cell migration guidance [21]. To investigate whether TH1 phosphorylation by c-Src participates in cell migration, we applied Transwell cell migration assay [21]. Consistent with our previous results, TH1 over-expression inhibited HeLa cell migration (Figure 6A). Compared with TH1-WT, the dephosphorylated form of TH1, TH1-Y6F, showed increased inhibition on c-Src (Y527F) driven HeLa and MCF-7 cell migration (Figure 6. B–C). Next we transfected TH1-Y6E (the constitutive phosphorylation form of TH1) into HeLa, measured the impact of TH1-Y6E on migration. Figure 6D demonstrated that TH1 mediated inhibition on cell migration was abolished by Y6E mutation. Taken together, these data indicated that Tyr-6 phosphorylation of TH1 led to enhanced cell migration.

### Induced Tyrosine Phosphorylation of TH1 by EGF and Estrogen Stimulations

**Figure 7:**
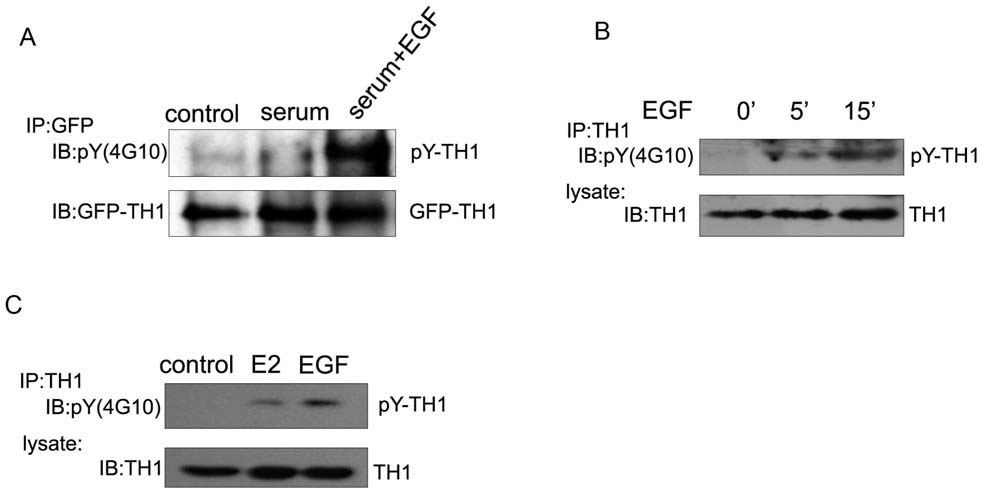
Induced Tyrosine Phosphorylation of TH1 by EGF and Estrogen Stimulations. (A) Induced tyrosine phosphorylation of TH1 by EGF in HEK293T cells. HEK293T cells over-expressing GFP-TH1 were serum starved for 18 h, pretreated with 50 µM pervanadate for 15 min and incubated with or without 100 ng/ml EGF for 15 min in the presence of 10% fetal bovine serum. Cell lysates were immunoprecipitated with antibody against GFP. The tyrosine phosphorylation of TH1 was analyzed with pY (4G10). The same blot was striped and re-probed with anti-GFP antibody. (B) Induced tyrosine phosphorylation of TH1 by EGF in A549 cells. A549 cells were serum starved for 18 h, pretreated with 100 µM pervanadate for 15 min, stimulated with 100 ng/ml EGF for 5 min and 15 min in the presence of 10% fetal bovine serum. Cell lysates were immunoprecipitated with TH1 antibody. The tyrosine phosphorylation of TH1 was analyzed with pY (4G10). (C) Induced tyrosine phosphorylation of TH1 by Estrogen and EGF in MCF-7 cells. MCF-7 cells were serum and estrogen deprived, pretreated with 100 µM pervanadate for 15 min, and stimulated with10 nM 17β-estradiol (E2) for 2 min or 100 ng/ml EGF for 15 min in the presence of 10% fetal bovine serum. Tyrosine phosphorylation of TH1 was analyzed as in (B).

To investigate the regulation of TH1 phosphorylation, we transfected GFP-tagged TH1 expression vector into HEK293T cells, serum starved for 18 h, and then stimulated with serum or serum plus EGF for 15 min. After stimulation, tyrosine phosphorylation of TH1 was measured. Interestingly, EGF plus serum induced tyrosine phosphorylation of TH1 significantly, whereas serum alone was not sufficient to induce tyrosine phosphorylation of TH1 (Figure 7A). Meanwhile, A549 cells exhibited significant tyrosine phosphorylation of endogenous TH1 by EGF treatment (Figure 7B). We next treated serum and estrogen starved MCF-7 cells with estrogen for 2 min or EGF for 15 min. Both stimuli induced rapid tyrosine phosphorylation of TH1 (Figure 7C).These results indicate that TH1 serves as a downstream molecule for EGF and estrogen receptor signaling, and its phosphorylation may play a role in EGF and estrogen receptor signaling.

## Discussion

The c-Src/Ras/ERK signaling pathway plays key role in the regulation of cell migration, which is orchestrated at multi-levels [4], [28], [29], [30], [31]. As an upstream kinase, c-Src phosphorylates multiple substrates and activates MAPK/ERK cascade [1], [28], [32], [33]. In this study we show that c-Src interacts with and phosphorylates TH1 at Tyr-6. Phosphorylation of TH1 decreases its association with A-Raf and PAK1, further decreases its inhibition on MAPK/ERK signaling. Moreover, Tyr-6 phosphorylation of TH1 enhances c-Src mediated cell migration, while TH1-Y6F, the dephosphorylated form of TH1, shows increased inhibition on c-Src mediated cell migration. The present data identify a novel mechanism whereby c-Src promotes TH1 phosphorylation, impacts its stability and decreases its inhibition on MAPK/ERK signaling.

Our previous studies have demonstrated that TH1 serves as an interaction partner and a negative regulator of A-Raf and PAK1, inhibiting MAPK/ERK signaling transduction [21], [22], [23]. Additionally, TH1 interacts with MEK1 and ERK1/2 without suppressing their kinase activities directly [21]. In contrast, numerous studies have highlighted c-Src as an important activator of MAPK/ERK signaling. Activated EGFR family receptor tyrosine kinases (RTKs) recruit and activate c-Src, and c-Src in turn further activates RTKs, stimulating MAPK/ERK signaling [4], [34]. c-Src is also activated by ligand-bound estrogen receptor (ER), which stimulates rapid, transient recruitment of c-Src and activates MAPK signaling [35]. In addition, the MAPK/ERK cascade is a well-known target of FAK–Src signaling [29], [36], [37], [38]. The present study further reveals the mechanisms of c-Src activating MAPK/ERK pathway. Spatial and temporal phosphorylation of TH1 induced by EGF and estrogen might participate in coordinate regulation of Ras/Raf/MEK/ERK signaling by c-Src. Through phosphorylation of TH1 at Tyr-6, c-Src suppresses inhibitory role of TH1 in MAPK/ERK pathway to further enhance its own role in activating MAPK/ERK signaling.

In addition to MAPK/ERK pathway, c-Src also regulates AKT and JNK pathways. c-Src activates PI3-K and Akt in response to GDNF [39], TRANCE [40] and VEGF [41], activates JNK pathway under many stimuli, including saturated fatty acids [42] and endothelin-1 [43]. Our group have shown that overexpression or knockdown of TH1 had little effect on the phosphorylation of Akt [24]. Whether c-Src effects on AKT and JNK pathways were involved with TH1 needs further study.

Our previous results indicated that c-Src enhanced cell migration through phosphorylation of TH1. Tyr-6 phosphorylation of TH1 led to decreased inhibition on cell migration, while TH1-Y6F, the dephosphorylated form of TH1, shows increased inhibition on c-Src mediated cell migration. The cell migration is an integrated process that requires the coordinated regulation of various molecules, including kinases, phosphatases, scaffold proteins and cell skeleton proteins [33], [44]. During the process, c-Src/Ras/ERK pathway plays key role, mediating phosphorylation of FAK, paxillin and p130Crk-associated substrate (CAS), recruiting key molecules to adhesion sites and regulating organization of actin cytoskeleton [29], [30], [32], [37], [45]. In response to the activation of v-Src, ERK was reported to be activated and targeted to newly forming focal adhesion, promoting migration through phosphorylating multiple substrates, including MLCK, FAK, paxillin, and calpain, which localize in focal adhesions and direct regulate turnover of focal adhesions and cytoskeleton organization [30], [46], [47], [48], [49], [50], [51]. Localized in focal adhesions and filopodia at the leading edge of cells, TH1 inhibits MAPK/ERK-driven cell migration through repressing the activation of MAPK/ERK pathway, and impacting actin and adhesion dynamics [21], [31]. Thus, Tyr-6 phosphorylation of TH1 by c-Src further extends our understanding on the role of c-Src in cell migration. Interestingly, the stability effect of TH1 phosphorylation by c-Src demonstrated in Fig. 5 did not show up in other figures. The possible explanation is that we used pervanadate, a protein tyrosine phosphatase inhibitor, to prevent TH1 dephosphorylation in Fig. 5. The phosphorylated TH1 may have lowered stability and rapid degradation. In the other experiments, we did not use pervanadate, allowing rapid dephosphorylation of phosphorylated TH1 by phosphatase. There may be a phosphorylation-dephosphorylation balance impacting TH1 stability.

Previous study by Chu *et al*. demonstrated that phosphorylation of p27 by c-Src impaired its Cdk2 inhibitory action and reduced its steady-state binding to Cyclin E-Cdk2 to facilitate Cyclin E-Cdk2-dependent p27 proteolysis, serving as an alternative way for c-Src promoting cell cycle progression [52]. Interestingly, they found that that c-Src-activated breast cancer cell lines showed reduced p27 expression and observed a correlation between c-Src activation and reduced nuclear p27 in primary human breast cancers [52]. Similar to their results, we found that phosphorylation of TH1 by c-Src showed a decreased association with A-Raf and PAK1, and a decreased inhibition on MAPK/ERK signaling and cell migration. In addition, our data revealed that estrogen stimulation induced tyrosine phosphorylation of TH1 in human breast cancer cells (Figure 7C). Given our previous observation that TH1 expression was decreased in aggressive breast cancer cell lines, and was negatively correlated to human breast tumor stage, especially lymph node metastasis [24], the potential role of phosphorylation of TH1 by c-Src in human breast cancers warrants further investigation.

In addition to its role in negative regulation of MAPK signaling, TH1 is an integral subunit of the human negative elongation factor (NELF) complex [18], [53]. When recruited to estrogen receptor-responsive promoters, NELF complex acts to stall RNAPII movement and attenuate estrogen receptor-dependent transcription [20]. TH1 is the first subunit of NELF complex being regulated by tyrosine phosphorylation. Furthermore, we observed that NELF complex assembly was abnormal in the presence of constitutive active c-Src (Wu *et al.,* unpublished data), suggesting possible role of c-Src in transcription elongation regulation.

In summary, the present study demonstrates that c-Src interacts with and phosphorylates trihydrophobin 1. The Tyr-6 phosphorylation of TH1 decreases its inhibition on MAPK/ERK signaling. Moreover, Tyr-6 phosphorylation of TH1 enhances c-Src mediated cell migration. Thus, phosphorylation of TH1 by c-Src may serve as an alternative way for c-Src promoting MAPK/ERK signaling and cell migration.

## Materials and Methods

### Reagents and Antibodies

Leupeptin, aprotinin, phenylmethylsulfonylfluoride, PP2, 17β-estradiol were purchased from Sigma-Aldrich Inc (St. Louis, MO, USA). Antibodies against ERK1/2, p-ERK 1/2 (Thr-202/Tyr-204), PAK1, GST and HA were purchased from Santa Cruz Biotechnology (Santa Cruz, CA, USA). Antibodies against c-Src and phospho-Src (Tyr-416) were purchased from Cell Signaling Technology (Danvers, MA, USA). Anti-Phosphotyrosine (4G10) antibody was purchased from Millipore (Bedford, Mass, USA). Antibodies against paxillin were purchased from Abcam. Antibodies against Myc and pcDNA3.1 vector were purchased from Invitrogen (Carlsbad, CA, USA). Protein G-agarose and anti-GFP antibody were products of Roche Applied Science (Mannheim, Germany). TH1 antiserum was described in previous studies [21], [23], [24]. Other reagents were commercially available in China.

### Cell Culture and Transfection

HEK293T, HeLa, COS-7, MCF-7, and A549 cell lines were obtained from the Institute of Cell Biology Academic Sinica (Shanghai, China). HEK293T, HeLa, and COS-7 cells were maintained in Dulbecco's modified Eagle's medium (DMEM) containing 10% fetal bovine serum (FBS) and 1% penicillin/streptomycin at 37°C in 5% CO_2_. MCF-7 breast cancer cells were cultured in 10% FBS–DMEM and supplemented with 0.01 mg/ml insulin, and A549 lung cancer cells were cultured in RPMI-1640 medium supplemented with 10% FBS. Cell transfection was carried out using Lipofectamine 2000 (Invitrogen, Carlsbad, CA, USA) according to manufacture's instructions.

### Plasmids Construction

Myc-tagged full length TH1 in pcDNA3.1 vector and pEGFP-N3 vector containing a GFP tag were described in previous studies [21], [22]. Full length wild type c-Src, constitutive active c-Src (Y527F) and kinase-dead c-Src (K298R) with or without HA-tag were constructed into pcDNA3 as previously described [25]. TH1 mutants were generated through replacement of specific TH1 tyrosine residue for phenylalanine by using a ‘bridge’ PCR method as described previously [54]. The amplified products were subcloned into pCDNA3.1 or PGEX-4T-1 vector. Mutagenic primers used for TH1 include, TH1-Y431F Forward: 5′- TCAGAACCAAGGTTCTTTCAGCTGC-3′ and Reverse: 5′- TCTGCAGCTGAAAGAACCTTGGTTC-3′, Y175F Forward: 5′-ATTTCTGACGCAGGGTTCCAGGGGG-3′ and Reverse: 5′- CTGGTGATCTCCCCCTGGAACCCTG-3′, Y5F Forward: 5′- ATGGACGAGGACTTCTACGGGAGCGCGG-3′ and Reverse: 5′- GAGGATCCGTTCACCATGATGAAG -3′,Y6F Forward: 5′-ATGGACGAGGACTACTTCGGGAGCGCGG-3′ and Reverse: 5′- GAGGATCCGTTCACCATGATGAAG -3′,Y5/6F Forward: 5′- ATGGACGAGGACTTCTTCGGGAGCGCGG-3′ and Reverse: 5′- GAGGATCCGTTCACCATGATGAAG -3′, The double mutant TH1 Y6/431F was generated by sequentially using the single mutant TH1 Y431F as Template. All the constructs were confirmed by sequencing.

### Immunoprecipitation Assay

Immunoprecipitation assay was performed as previously described [21], [22]. Briefly, 36 h after transfection, cells were lysed with 0.5 ml of IP lysis buffer (50 mM Tris-HCl, pH 7.5, 150 mM NaCl, 0.1% Nonidet P-40, 5 mM EDTA, 5 mM EGTA, 15 mM MgCl_2_, 60 mM beta-glycerophosphate, 0.1 mM sodium orthovanadate, 0.1 mM NaF, 0.1 mM benzamide, 10 µg/ml aprotinin, 10 µg/ml leupeptin, 10 µg/ml phenylmethylsulfonyl fluoride) for 1.5 h at 4°C. The whole cell lysates were incubated first with indicated antibody at 4°C for 1 h and then with protein G-agarose beads at 4°C overnight. The bound proteins were eluted by boiling in SDS sample buffer and detected by western blot assay.

### Western Blot Assay

Western blot experiments were performed as previously described [21], [22]. Briefly, the cells were lysed in SDS lysis buffer (40 mM Tris pH 7.4, 150 mM NaCl, 1 mM EDTA, 1% SDS, 1 mM aprotinin, 1 mM PMSF and 10 µg/ml leupeptin). Cell lysates were separated by electrophoresis in 8% or 10% SDS-PAGE and then transferred to a PVDF membrane (Roche Applied Science). After blocking with 5% nonfat milk, the membrane was incubated with indicated antibody and then developed using the enhanced chemiluminescent (ECL) assay kit.

### Immunofluorescence Assay

Cells were processed for immunofluorescence assay as described previously [21], [22]. The antibodies used here were rabbit anti-TH1 (1∶200) antibody, FITC-conjugated goat anti-rabbit IgG (1∶200) secondary antibody, mouse anti-c-Src (1∶100) antibody and Rhodamine-conjugated goat anti-mouse IgG (1∶200) secondary antibody. The cells were mounted on glass slides using Fluoromount mounting medium (Sigma-Aldrich, St. Louis, MO). The images of stained cells were captured using a Leica TCS SP5 confocal microscope.

### GST-Pull Down Assay and Expression of Recombinant Proteins

The recombinant GST-tagged wild type TH1 and mutants were expressed by isopropyl-thiogalactoside (IPTG) induction of *Escherichia coli* containing the corresponding pGEX-4T-1 constructs, purified by Glutathione-Sepharose 4B beads according to the manufacture's instruction (GE Healthcare life science). As for GST-pull down assay, purified GST-tagged proteins (5 µg) were incubated with HeLa cell lysates, precipitated by glutathione-Sepharose 4B beads. The unbound proteins were removed by washing the beads three times with IP lysis buffer, whereas retained proteins were resolved by SDS-PAGE and analyzed by Western blot.

### Phosphorylation Assay

Thirty-six hours after transfection, cells were trypsinized and washed three times with ice-cold PBS. Cell pellets were suspended in RIPA buffer (150 mM NaCl, 1% NP- 40, 0.5% deoxycholate, 0.1% SDS, 50 mM Tris–HCl, pH 7.5) with additional 1% SDS and boiled for 10 min to disrupt protein–protein interaction. The lysates were further diluted with ten volumes of RIPA buffer, sonicated on ice, clarified by centrifugation, pre-cleared with Protein-G agarose for 45 min at 4°C and immunoprecipitated with indicated antibody. The immunoprecipitated proteins together with whole cell lysates (40 µg) were analyzed by western blots using anti-Phosphotyrosine (4G10) antibody.

### In vitro Kinase Assay

Purified GST, GST-TH1 or GST-TH1 mutant (5 µg) was incubated with recombinant human c-Src (5 U, from Millipore, Bedford, MA) in 30 µl kinase buffer (10 mM HEPES, pH 7.5, 150 mM NaCl, 2.5 mM DTT, 0.01% Triton X-100, 10 mM MnCl_2_, 100 µM ATP) for 30 minutes at 30°C. The reaction was stopped by adding SDS sample buffer and boiling for 5 min. Samples were subjected to SDS-PAGE on 8% gels and transferred onto PVDF membranes for western blot using anti-Phosphotyrosine (4G10) antibody.

### Transwell Cell Migration Assay

Transwell assay was performed as described previously [21] using Boyden chambers (tissue culture-treated, 6.5-mm diameter, 10-µm thickness, 8-µm pores from Costar Corp, Cambridge, MA) containing polycarbonate membranes. Briefly, serum starved HeLa cells were trypsinized and counted. Then 100 µl of 10^6^ cells in serum-free medium were added to the upper chamber and 500 µl of the appropriate medium with 10% FBS was added to the lower chamber. The Transwell was incubated for 18 h at 37°C. Non-migratory cells on the upper membrane surface were removed with a cotton swab, and the migratory cells on the undersurface of the membrane were fixed and stained with 0.1% crystal violet for 20 min at room temperature. Photographs of 6 random regions were taken, and the number of cells was counted to calculate the average number of migrated cells.
